# The Relationship between Nomophobia, Insomnia, Chronotype, Phone in Proximity, Screen Time, and Sleep Duration in Adults: A Mobile Phone App-Assisted Cross-Sectional Study

**DOI:** 10.3390/healthcare11101503

**Published:** 2023-05-22

**Authors:** Haitham Jahrami

**Affiliations:** 1 Government Hospitals, Manama P.O. Box 12, Bahrain; hjahrami@health.gov.bh; 2 Department of Psychiatry, College of Medicine and Medical Sciences, Arabian Gulf University, Manama P.O. Box 26671, Bahrain

**Keywords:** sleep, anxiety, addiction, smartphone, social media, circadian rhythm

## Abstract

Earlier studies that have investigated the association between nomophobia and insomnia revealed that a strong relationship exists between both variables. This study aimed to explore possible associations between these variables and their impact on physical and mental health outcomes using a cross-sectional study design and mobile phone apps to collect data. Using a survey approach, data were collected from 444 participants (52% female, mean age 34 ± 12) using the Nomophobia Questionnaire (NMP-Q), the Insomnia Severity Index (ISI), the Morningness–Eveningness Questionnaire (MEQ) and three Android mobile phone apps. The Plees Tracker, screen time, and pedometer apps aided in collecting data on sleep duration, time spent on screen per day, and how close the phone was to the person. A statistically significant association was noted between nomophobia and insomnia, nomophobia and the eveningness chronotype, and nomophobia and screen time. The eveningness chronotype was also associated with an increased screen time use. The results show that NMP-Q, ISI, and screen time increase according to the chronotype. No statistically significant differences were noted in daily steps or sleep duration according to chronotype. The findings suggest that interventions targeting nomophobia may be beneficial in addressing insomnia among adults, particularly those with an evening chronotype. Future studies should consider exploring the causal relationship between them.

## 1. Introduction

The fear or anxiety of not having a mobile phone or not being able to use one is known as nomophobia, which is an acronym for “NO MObile PHone PhoBIA” [1]. Nomophobia has become a growing concern in contemporary society as a result of the quick rise in mobile phone usage, particularly among young adults [1,2]. The prevalence of insomnia, a common sleep disorder marked by difficulty falling or staying asleep, has also increased recently [3]. Previous studies that looked into the association between nomophobia and insomnia revealed that a strong relationship exists between both variables [3,4,5,6,7]. Numerous studies have shown a connection between excessive mobile phone use, particularly at night, and sleep issues such as insomnia and shorter sleep durations by an average of 60 min [8,9,10,11]. In Saudi Arabia, recent research demonstrated that a smartphone was owned by more than 98% of the respondents [8]. The same study documented that 90% of people use their smartphones before going to sleep, and the most popular service among participants was social media [8].

Another element that may affect the link between nomophobia and insomnia is chronotype, or a person’s innate preference for wake and sleep times [12]. Morning, intermediate, and evening chronotypes are the broad categories for chronotypes [12]. Evening chronotype people are more likely to have sleep issues and insomnia because they prefer to be awake and active later into the night [13]. However, little research has been performed on the relationship between chronotype, nomophobia, and insomnia. According to recent research, using a mobile phone before bed can have a negative impact on how well adults sleep [14]. The research concluded that given how quickly and significantly the capabilities of mobile phones are evolving, it merits continuing scientific attention [14].

Another research work investigated the frequency and average length of in-bed media (i.e., smartphone and tablets) use reported by the respondents, along with their symptoms of insomnia, daytime sleepiness, preference for the morning or evening, and bedtime and rise times on their off days [11]. The average nightly media use time was 46.6 min [11]. The findings revealed a positive correlation between computer use for reading, playing, and surfing and a negative correlation between morningness and sleepiness [11]. Insomnia and chronotype were positively and adversely correlated with mobile phone use for playing, surfing, and texting, while morningness was negatively correlated [11]. No other media platforms or media usage in general was connected to either of these factors or daytime sleepiness [11].

Self-reporting via questionnaires served as the main method of data collection in earlier studies on subjects such as nomophobia, insomnia, and chronotype [1,2]. This shows that questionnaires and questions about participants’ experiences, actions, and attitudes about these subjects were given to them. Utilizing only self-report measures, however, has some drawbacks. Participants might not always accurately recall or describe their experiences, for instance, or they might not feel comfortable disclosing certain details. Self-reporting measures also lack objective data or measurements, which can affect the reliability and accuracy of findings. On the other hand, objective measurements are founded on precise observations or measurements of a phenomenon. Tracking smartphone usage (i.e., screen time) or physiological reactions to use or separation from a mobile phone (i.e., steps taken with a phone in proximity) may be considered as objective measures of nomophobia. Earlier studies on nomophobia, insomnia, and chronotype may not have been able to fully capture these phenomena because they only used self-reporting measures. It is obvious that future studies could benefit from including objective metrics to help them better understand these problems. This cross-sectional study investigated the association between chronotype, insomnia, and nomophobia in adults.

## 2. Materials and Methods

### 2.1. Design

The study aimed to explore possible associations between these variables and their impact on physical and mental health outcomes using a cross-sectional study design and mobile phone apps to collect data.

### 2.2. Selection Criteria and Sample Size

Adults aged 18 years and more were recruited through online platforms and social media. Participants were selected using convenience sampling and had to be (1) free from any known medical or psychiatric conditions, (2) able to supply informed consent, and (3) an Android mobile phone user to be eligible for the study.

The sample size was decided using the following formula: n = Z^2^ × p × (1 − p)/d^2^, where n is the sample size; Z is the z-score that corresponds to the desired confidence level (for example, 1.96 for a confidence level of 95%); *p* is the expected prevalence of the outcome of interest; (1 − p) is the complement of the expected prevalence; *p* is the expected prevalence of the outcome of interest; and d is the desired level of precision (i.e., the maximum allowable margin of error) [15]. Based on the calculations, it was estimated that a sample size of at least 385 participants was needed to achieve the desired level of precision of 5% and a confidence level of 95%.

### 2.3. Participants

Participants for our research study were drawn from the general population of people aged 18 and above in all regions of Bahrain (i.e., Capital, Muharraq, Northern and Southern). To recruit participants from multiple online platforms and social media channels, the study used a convenience sample strategy. A total of 444 participants (52% females) were included in the final analyses. The mean age of the study participants was 34 ± 12 years.

### 2.4. Measures

#### 2.4.1. Self-Reporting Measures

Socio-demographic data

In this research study, socio-demographic data from study participants were collected. The variables included age, gender, marital status, and occupation. This information was collected through self-reporting measures. Using self-reported anthropometric measurements, the study participants’ physical characteristics were evaluated. This involved gathering data on each participant’s weight and height, which were then used to calculate their body mass index (BMI).

Nomophobia

The Nomophobia Questionnaire (NMP-Q) developed by Yildirim and Correia was used to gauge the severity of nomophobia among the participants [16]. The questionnaire is graded on a 7-point Likert scale, with higher scores indicating greater nomophobia [16]. The NMP-Q is a multifactor measure that consists of four factors: “Factor 1: not being able to communicate”, “Factor 2: losing connectedness”, “Factor 3: not being able to access information”, and “Factor 4: giving up convenience” [16]. The sum of all factors is interpreted as 20–59: mild nomophobia; 60–99: moderate nomophobia; and 100–140: severe nomophobia [16]. In the present study, the Arabic language version adaptation by Al-Balhan and colleagues was used [17].

Several studies have investigated the NMP-Qs psychometric qualities [18]. Overall, the questionnaire has proven to be reliable and valid across a wide range of groups and cultures [18]. The NMP-Q has a strong internal consistency, with Cronbach’s alpha coefficients ranging from 0.87 to 0.94 in several investigations [18]. In the present study, the Cronbach’s alpha for the NMP-Q was 0.94. The NMP-Q has also shown strong test–retest reliability, with correlation values ranging from 0.81 to 0.89 over a two- to four-week period [19,20].

The NMP-Q has strong construct validity, as it correlates with other measures of problematic mobile phone usage, such as the Mobile Phone Problematic Usage Scale (MPPUS) [21] and the Mobile Phone Dependence Questionnaire (MPDQ) [22]. The NMP-Q has also shown strong convergent validity, since it correlates with measures of anxiety, depression, and stress [18]. The NMP-Q has good discriminant validity because it correlates with personality trait measures such as extraversion and neuroticism [20,23].

Insomnia

The severity of insomnia symptoms among the participants was assessed using the Insomnia Severity Index (ISI) which was developed by Morin [24]. The ISI is a seven-item self-report questionnaire scored on a Likert scale with a maximum of five points; higher scores indicate more severe insomnia [24,25]. The ISI is interpreted as 0–7: no clinically significant insomnia; 8–14: subthreshold insomnia; 15–21: clinical insomnia (moderate severity); and 22–28: clinical insomnia (severe severity) [24,25]. The ISI is a unifactor scale [25]. In the present study, the Arabic language version which was adapted by Suleiman and Yates was used [26].

The ISIs psychometric qualities have been thoroughly researched, and it has been found to be a reliable and valid instrument for assessing the severity of insomnia [25]. The ISI has been proven to have strong internal consistency in various investigations, with Cronbach’s alpha coefficients ranging from 0.74 to 0.91 [25]. In the present study, the Cronbach’s alpha of the ISI was 0.96.

The ISI has also exhibited strong test–retest reliability, with correlation coefficients ranging from 0.76 to 0.96 over a one- to four-week period [25]. The ISI has been determined to have strong construct validity because it corresponds with other measures of insomnia severity such as polysomnography and actigraphy [27]. The ISI has been shown to be sensitive to changes in the intensity of insomnia after therapy [25]. The ISI has been translated into multiple languages and validated in various cultures, making it a helpful tool for assessing the severity of insomnia in diverse groups [25]. It is widely used in clinical practice and research to assess the severity of insomnia symptoms and to track treatment effects.

Chronotype

To determine the participants’ chronotype, the Morningness–Eveningness Questionnaire (MEQ) [28], which was developed by Horne and Östberg in 1976, was used. The MEQ is a unifactor questionnaire with 19-item self-report questions [28]. High scores indicate morningness type and low scores indicate eveningness type [28]. Specifically, the MEQ is interpreted as below 42: evening preference; between 42 and 58: intermediate preference; and above 58: morning preference [28]. In the present study, the Arabic language version which was translated by Al-Owaisia and colleagues was used [29].

The MEQs psychometric qualities have been widely researched, and it has been determined to be a trustworthy and valid instrument for determining chronotype. In several investigations, Cronbach’s alpha coefficients ranged from 0.77 to 0.88, indicating that the MEQ has strong internal consistency [30,31]. In the present study, the Cronbach’s alpha for the MEQ was 0.90.

The MEQ has also shown strong test–retest reliability, with correlation values ranging from 0.75 to 0.90 over a two- to ten-week period [30,31]. The MEQ has strong construct validity, as it corresponds with other chronotype measures, such as the Munich Chronotype Questionnaire [32,33]. The MEQ has been translated into multiple languages and validated in various cultures, making it an effective tool for determining chronotype in diverse groups [30,31,32,33]. It is frequently used in studies to study the association between chronotype and other health outcomes such as sleep quality, mood, and cognitive performance [30,31,32,33].

#### 2.4.2. Objective Measures

Three open-source apps which are software applications whose source code is freely available and can be changed and distributed by anyone were used in this research. Open-source apps are often used in research studies as a cost-effective and flexible tool for collecting data or conducting experiments. Furthermore, open-source apps can be customized to suit the specific needs of a research study, and they can be changed to include added features or functionality [34]. They can also be easily shared with other researchers or participants, which can increase the transparency and reproducibility of research findings. The apps are built and tested on Android operating systems only and this explains why inclusion criteria emphasize that participants use Android devices as their primary device.

Sleep duration

A sleep tracking program for Android smartphones called “Plees Tracker” was used to objectively collect bedtime, wake-up time, and sleep duration. The app is a straightforward tool that enables users to check their sleep duration and track their sleeping patterns over time. Users add details to the app, such as their bedtime and wake-up time, to help the sleep tracking data be more exact. Users of the app can also access a sleep duration score, which is found by the quantity of their sleep. Plees Tracker is a free, simple, and user-friendly sleep-tracking app that can be useful for individuals to learn more about their sleep patterns and improve their sleep habits. The app was developed by Miklos Vajna; the source code is available at https://github.com/vmiklos/plees-tracker, accessed on 1 October 2022.

Screen time

An application called “screen time” was made to help people keep track of and regulate how much time they spend using tablets and smartphones. The use of the screen time app was also a helpful tool in showing a person’s morning–evening chronotype. Chronotype is the term used to describe a person’s preferred sleep and wakefulness patterns. It can be classified as morning, intermediate, or evening depending on when a person is at their most active and alert. The app was developed by Markus Fisch; the source code is available at https://github.com/markusfisch/ScreenTime, accessed on 1 October 2022.

Physical activity/phone in proximity

An application called “pedometer”, a physical activity app, was made to track a person’s level of physical activity, particularly the number of steps they take each day. The application counts the number of steps taken by detecting movement using the sensors on a smartphone or wearable device such as an accelerometer. The app was developed by j4velin development; the source code is available at https://github.com/j4velin/Pedometer, accessed on 1 October 2022.

In this study, the pedometer app was used to measure “phone in proximity”, which refers to the state in which a mobile phone is physically close to an individual or within their immediate vicinity.

### 2.5. Data Collection Procedure

**Figure 1:**
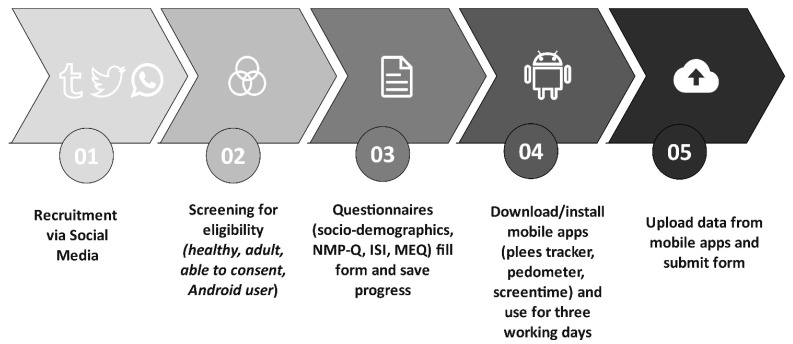
Flow diagram of the study.

Utilizing an online survey platform, data were gathered. Before completing the questionnaires, participants gave their informed consent. Participants in the study had the choice of manually entering their results or using the export feature to upload the unprocessed data from their activity tracking apps. Participants had to keep track of their screen time, pedometer steps, and sleeping habits for three straight working days as part of the study’s protocol. The three apps’ default modes automatically computed the means to help participants complete their tasks. The data collection flowchart is shown in Figure 1.

### 2.6. Ethics

The Institutional Review Board gave its approval for the study. The Declaration of Helsinki’s ethical guidelines were followed in the research study for the collection of all data. All participants gave their free, explicit consent; the researchers safeguarded the privacy of their information; and took measures to ensure the security and welfare of vulnerable groups. Adherence to the Declaration of Helsinki is crucial to achieving this goal because the researchers are committed to upholding ethical standards and making sure that the research is conducted in a responsible and respectful manner. The protocol was reviewed and approved by the Research Ethics Committee of the Ministry of Health in Bahrain, approval code: SHCRC/29/09/2022.

### 2.7. Data Analysis

Prior analysis data were visually assessed using histogram charts to see whether the data obtained for a certain variable had a normal distribution. By examining the shape of the histograms, the data appeared to be normally distributed and therefore parametric statistics were computed and reported.

R statistical software was used to analyze all the data in the study. A variety of statistical tests and models can be run on the data using R, a robust and popular statistical analysis software program. The *p*-value was set at 0.05, which is a standard cutoff for deciding statistical significance. A *p*-value of 0.05 means that there is a 5% chance that the results were the result of chance. The *p*-value shows the likelihood that the observed results were the result of chance.

For the sample, descriptive statistics were computed and Pearson’s correlation was used to look at the connection between study variables. A one-way analysis of variance (ANOVA) was used to find whether there were significant differences between the means of the study variables according to chronotype (i.e., morningness, intermediate, and eveningness). Following the one-way ANOVA, post hoc testing was used to identify which groups varied substantially from one another. Tukey’s Honestly Significant Difference (HSD) [35] test was employed to perform the post hoc testing. This test is a standard statistical analysis method for comparing multiple means and is regarded as a conservative approach to discovering significant differences between groups. The Tukey HSD test evaluates all possible pairs of groups mean differences and determines if they are statistically significant [35]. Post hoc testing is a critical stage in statistical research since it identifies which individual groups differ significantly from one another. Post hoc testing enable researchers to establish the specific differences between groups that are statistically significant and might be regarded important by utilizing the Tukey HSD test [35].

## 3. Results

Table 1 shows the descriptive statistics of the participants in the study. All the participants had some level of nomophobia. Specifically, mild, moderate, and severe nomophobia was suffered by 6%, 70%, and 24% of the respondents, respectively. Insomnia was common too, with 54% meeting the insomnia criteria according to ISI. Most of the participants (70%) were of intermediate chronotype, while 11% were of morningness type and 19% were of eveningness type.

In the present study, about 50% of the sample were students, and it was interesting to investigate whether their employment status had any influence on the relationship between these variables. A one-way ANOVA revealed that employment status (i.e., students vs. employed vs. non-employed) did not affect the distribution of the results of all *p*-values > 0.3.

Table 2 shows a correlation matrix of the study variables. A statistically significant association was noted between nomophobia and insomnia, nomophobia and eveningness chronotype, and nomophobia and screen time. The eveningness chronotype was also associated with an increased screen use time. The results showed a statistically significant correlation was obtained between NMP-Q and ISI, *r* = 0.32, *p* = 0.001; NMP-Q and MEQ, *r* = −0.49, *p* = 0.001; NMP-Q and screen time, *r* = 0.11, *p* = 0.02; ISI and MEQ, *r* = −0.36, *p* = 0.001; and finally MEQ and screen time, *r* = −0.14, *p* = 0.003.

Table 3 shows the differences in the study variables according to chronotype using ANOVA. The results show that NMP-Q, ISI, and screen time increase according to chronotype. No statistically significant differences were noted in daily steps or in sleep duration according to chronotype.

Table 4 shows the Tukey HSD post hoc comparison for the study variables according to chronotype. Statistically significant differences were obtained for the NMP-Q, ISI, and screen time. According to Table 4, the NMP-Q and ISI scores were highest for evening, intermediate, and morning chronotypes, respectively. Screen time was higher for the evening chronotype compared to morning and intermediate chronotypes by approximately 50 and 40 min, respectively.

## 4. Discussion

This study aimed to investigate the relationship between nomophobia, insomnia, chronotype, phone in proximity, screen time, and sleep duration in adults. The findings revealed a significant positive correlation between nomophobia and insomnia, supporting earlier research showing that excessive mobile phone use may contribute to sleep disturbances. Furthermore, the results showed that evening chronotype individuals had higher nomophobia and insomnia scores compared to those with morning or intermediate chronotypes. The most important and novel finding of this research is that screen time is a better marker for nomophobia compared to step count (i.e., merely carrying the phone around). Digital biomarkers or mobile health (mHealth) biomarkers are usual terms used to describe markers produced from mobile apps [36]. These biomarkers are created using information gathered by sensors and other technology found in mobile devices, including smartphones or wearables [36]. According to the findings of the present study, digital biomarkers that serve as indicators of health outcomes and can be used to diagnose and track a variety of chronic diseases and ailments can be created from data from Biometric Monitoring Technologies (BioMeTs) [36], including mHealth and wearables.

Nomophobia, or the fear of living without a phone, is becoming increasingly common in contemporary society. People have grown more dependent on their smartphones because of their widespread use for communication, entertainment, and even routine daily activities. However, sleep problems such as insomnia have also been connected to this dependence on technology. Unsurprisingly, in the present study, shorter sleep durations, more severe insomnia symptoms, and the evening chronotype were all associated with higher scores on the nomophobia scale. These results are in line with earlier studies showing how technology use interferes with sleep [8,37]. An overdependence on mobile devices that interferes with sleep-promoting habits and keeps one up at night worrying is reflected in the dread of missing out and compulsive phone checking behaviors [38]. One’s natural transition to sleep is probably delayed by alerts, light exposure, and engaging with stimulating content on smartphones [39]. It may be possible to reduce these correlations and advance improved sleep health by limiting phone use, keeping a regular sleep schedule, and improving sleep hygiene [39].

Stress, anxiety, or depression are common psychological causes of insomnia, which are characterized by difficulties in falling or staying asleep [40]. It has been established that using a mobile device right before bedtime increases the risk of insomnia [5]. According to a recent systematic review, the blue light that screens emit can interfere with the body’s normal sleep–wake cycle, making it harder to fall asleep and lowering the quality of one’s sleep [41]. Additionally, individuals may find it challenging to unwind and go to sleep due to the constant notifications and alerts from mobile phones, which can heighten anxiety [5].

Nomophobia can make insomnia worse because sufferers may experience panic or anxiety when they are away from their phone. People may spend more time on their phones to prevent withdrawal or anxiety, which can result in increased screen time [42]. A recent study found that people with higher levels of nomophobia also had lower sleep quality, as determined by the Pittsburgh Sleep Quality Index [43].

This association between nomophobia and insomnia may have important repercussions. Lack of sleep can cause a variety of physical and mental health issues, such as immune system problems [44], mood disorders [45], and an increased risk of developing chronic illnesses such as diabetes [46] and heart disease [47]. The constant use of mobile devices can also have an adverse effect on social interactions, resulting in a sense of loneliness and disconnection from others [48].

Several studies have suggested a link between mobile phone use, headaches, and sleep issues [49,50]. According to two studies, mobile phone use is connected with an increased risk of tension headaches and poor sleep quality [51,52]. Another study discovered that frequent mobile phone use was linked to greater pain sensitivity and poor sleep quality [53]. Excessive mobile phone use has also been associated with increased levels of insomnia, a lower sleep quality, and greater stress and worry [53]. While additional research is needed to fully understand the underlying mechanisms and potential therapies, these findings highlight the significance of restricting mobile phone use, particularly at night, in order to improve sleep quality and reduce the risk of headaches and other health concerns.

According to the findings of the current study, people with an evening chronotype score higher for nomophobia and insomnia than people with morning or intermediate chronotypes. This finding is significant because it clarifies the possible dangers of being a night owl in the digital age. This might be because evening chronotypes use their phones more often and for longer stretches of time, especially at night and in the evening, which can disturb sleep and amplify nomophobia symptoms.

The implications of this study suggest that individuals with an evening chronotype may be at greater risk for both nomophobia and insomnia and may receive help from targeted interventions to help mitigate these risks. For people with an evening chronotype, techniques such as creating a regular sleep schedule, limiting screen time before bed, and engaging in mindfulness or meditation may be especially beneficial. Interventions that aim to cut back on mobile phone use or encourage healthy digital habits may also be successful in lowering anxiety levels and dependence on technology [54].

Compared to steps taken or simply carrying a phone, screen time seemed to be a better indicator of nomophobia. This finding is significant because it emphasizes how important it is to consider the type and frequency of mobile phone use when evaluating nomophobia. This suggests that rather than simply carrying the phone or counting steps, the frequency and intensity of mobile phone use may be a more reliable predictor of nomophobia. By taking a more nuanced approach to measuring mobile phone use, this study supplies important insights into the complex relationship between nomophobia and technology.

Strengths and limitations

A major strength of this study is the use of mobile phone apps to collect data, which supplied a convenient and practical way of measuring variables such as screen time and phone proximity, which may have been difficult to measure using traditional self-report methods. Data collection using mobile phone apps gives a more objective and reliable measure of mobile phone use and screen time than self-reported measurements. By enabling continuous, real-time monitoring of a person’s health and enabling early diagnosis and intervention of health conditions, digital biomarkers have the potential to revolutionize healthcare.

Furthermore, the study collected data on a range of variables, including nomophobia, insomnia, chronotype, screen time, and physical activity, which allowed for a comprehensive analysis of the associations between these variables. The use of numerous measures, such as the NMP-Q, ISI, MEQ, and mobile phone apps, enables a thorough examination of the links between nomophobia, insomnia, and chronotype. The findings are more generalizable due to the adequate sample size of 444 people.

Some limitations need to be acknowledged. The cross-sectional design of the study does not allow for the determination of causality between the variables studied. The study relied on a convenience sample, which may limit the generalizability of the findings to other populations. The study only collected data from Android users, which may limit the generalizability of the findings to other users. The study did not collect data on other potential confounding factors, such as coffee use or anxiety, depression, or physical activity levels, which could have influenced the study results.

Implications for practice

The study’s conclusions have a number of practical ramifications, which are summarized in the following.

Healthcare practitioners should consider testing their patients for insomnia symptoms and nomophobia, especially if they have an evening chronotype. Sleep problems (i.e., insomnia) may be improved by interventions that focus on nomophobia. Therefore, in order to address nomophobia and enhance the insomnia symptoms or poor quality of sleep, healthcare practitioners may think about implementing interventions such as cognitive behavioral therapy, mindfulness-based interventions, or instruction on responsible mobile phone use [3]. Interventions to cut back on screen time may be advantageous for people with an evening chronotype given the correlation between that chronotype and higher use of screens [1,4]. These interventions may comprise establishing screen time restrictions, encouraging the use of blue light filters, or promoting non-screen time alternatives for nighttime activities [11]. Healthcare practitioners may find it helpful to screen and monitor patients with nomophobia and insomnia using mobile phone apps that collect data on sleep length, screen time, and proximity to phones in order to adapt therapies. Future research should examine the causal link between insomnia and nomophobia as well as the efficacy of different treatments of these problems.

Implications for future research

The research’s conclusions point towards numerous directions for future study, summarized in the following

The study’s cross-sectional methodology makes it difficult to determine whether nomophobia and sleeplessness are related. Future research should make use of longitudinal designs to examine potential causal pathways and the temporal link between these variables. The research identified an association between the eveningness chronotype and higher screen use; thus, future studies should look into the underlying mechanisms causing this association and consider potential screen time reduction strategies for people with evening chronotypes. The validity and reliability of these measurements should be investigated in more detail in the future, and it should also be determined whether it is practical and efficient to screen for and monitor insomnia and nomophobia using mobile phone apps. Finally, since the study only looked at adults, future studies should look into similar issues among other age groups, including teenagers and senior citizens who may be particularly susceptible to insomnia and narcolepsy.

## 5. Conclusions

The complex interactions between nomophobia, insomnia, chronotype, phone proximity, screen time, and sleep duration in adults are discussed in detail by this study, which offers significant new insights. The findings draw attention to the potential dangers of excessive mobile phone use, especially for people with evening chronotypes who may be more susceptible to nomophobia and insomnia.

Nomophobia and insomnia have a strong positive correlation, which is consistent with earlier studies on the effects of mobile phone use on sleep quality. Importantly, the study also discovered that screen time is a better indicator of nomophobia than step count, emphasizing the significance of taking specific mobile phone usage patterns into account when deciding the risk of nomophobia.

The findings suggest that interventions targeting nomophobia may be beneficial in addressing insomnia among adults, particularly those with an evening chronotype. Future studies should consider exploring the causal relationship between these two.

## Figures and Tables

**Table 1:** Descriptive statistics of the study participants (n = 444).

**Continuous Variables**	
Age, years	34 ± 12
Height, cm	168 ± 9
Weight, kg	70 ± 16
BMI, kg/m^2^	25 ± 5
NMP-Q	78 ± 19
ISI	12 ± 6
MEQ	50 ± 11
Steps/day	6121 ± 1545
Distance/day, km	5 ± 1
Screen Time/day, minutes	329 ± 68
Bedtime, HH:MM	23:13
Wake time, HH:MM	05:24
Sleep duration, HH:MM	06:10
Sleep duration/night, minutes	370 ± 33
**Categorical Variables**	
Sex (%)	
▪ Male ▪ Female	214 (48%) 257 (52%)
Marital status (%)	
▪ Married ▪ Single	158 (36%) 286 (64%)
Employment (%)	
▪ Employed ▪ Not employed ▪ Student	160 (36%) 62 (14%) 222 (50%)
Nomophobia category (%)	
▪ Mild ▪ Moderate ▪ Severe	28 (6%) 311 (70%) 105 (24%)
Insomnia category (%)	
▪ No insomnia ▪ Subthreshold insomnia ▪ Moderate clinical insomnia ▪ Severe clinical insomnia	161 (36%) 46 (10%) 229 (52%) 8 (2%)
MEQ category (%)	
▪ Morningness ▪ Intermediate ▪ Eveningness	48 (11%) 313 (70%) 83 (19%)

Note: The data are expressed as arithmetic means ± standard deviation or count (percentage %). NMP-Q = nomophobia questionnaire; ISI = insomnia severity index; MEQ = morningness–eveningness questionnaire.

**Table 2:** Correlation matrix of the study variables.

	NMP-Q	ISI	MEQ	Steps	Screen Time	Sleep Duration
NMP-Q	—					
ISI	*r =* 0.32 *p* = 0.001	—				
MEQ	*r =* −0.49 *p* = 0.001	*r =* −0.36 *p* = 0.001	—			
Steps	*r =* 0.01 *p* = 0.86	*r =* 0.07 *p* = 0.16	*r =* 0.03 *p* = 0.55	—		
Screen time (minutes/day)	*r =* 0.11 *p* = 0.02	*r =* 0.02 *p* = 0.74	*r =* −0.14 *p* = 0.003	*r =* 0.02 *p* = 0.73	—	
Sleep duration (minutes/day)	*r =* −0.08 *p* = 0.10	*r =* −0.01 *p* = 0.86	*r =* 0.06 *p* = 0.24	*r =* −0.05 *p* = 0.30	*r =* −0.06 *p* = 0.19	—

Note: The Pearson product–moment correlation coefficient. NMP-Q = nomophobia questionnaire; ISI = insomnia severity index; MEQ = morningness–eveningness questionnaire; —: Its standard presentation for matrix.

**Table 3:** Differences in the study variables according to chronotype.

Measure	Morning	Intermediate	Evening	F (df)	*p*-Value
NMP-Q	67 ± 10	73 ± 13	105 ± 16	153.33 (2)	0.001
ISI	10 ± 3	11 ± 5	17 ± 5	65.38 (2)	0.001
Steps	5770 ± 1424	6192 ± 1534	6058 ± 1641	1.84 (2)	0.164
Screen time	289 ± 66	330 ± 66	345 ± 67	11.27 (2)	0.001
Sleep duration	375 ± 37	370 ± 32	365 ± 33	1.37 (2)	0.26

Note: One-way analysis of variance. NMP-Q = nomophobia questionnaire; ISI = insomnia severity index; MEQ = morningness–eveningness questionnaire.

**Table 4:** Post hoc testing for the study variables according to chronotype.

Measure	Evening -Intermediate	Evening -Morning	Intermediate -Morning
NMP-Q	MD = 31, *p* < 0.001	MD = 38, *p* < 0.001	MD = 7, *p* < 0.007
ISI	MD = 6, *p* < 0.001	MD = 8, *p* < 0.001	MD = 2, *p* < 0.07
Steps	MD = −134, *p* < 0.80	MD = 288, *p* < 0.60	MD = 421, *p* < 0.20
Screen time	MD = 15, *p* < 0.17	MD = 52, *p* < 0.001	MD = 41, *p* < 0.001
Sleep duration	MD = −6, *p* < 0.38	MD = −10, *p* < 0.22	MD = −5, *p* < 0.70

Note: Tukey HSD post hoc test. NMP-Q = nomophobia questionnaire; ISI = insomnia severity index; MEQ = morningness–eveningness questionnaire. Results are expressed as mean differences (MD) and *p*-value.

## Data Availability

Datasets analyzed during the current study are available immediately upon request from the corresponding author.
